# Orientia, rickettsia, and leptospira pathogens as causes of CNS infections in Laos: a prospective study

**DOI:** 10.1016/S2214-109X(14)70289-X

**Published:** 2015-02

**Authors:** Sabine Dittrich, Sayaphet Rattanavong, Sue J Lee, Phonepasith Panyanivong, Scott B Craig, Suhella M Tulsiani, Stuart D Blacksell, David A B Dance, Audrey Dubot-Pérès, Amphone Sengduangphachanh, Phonelavanh Phoumin, Daniel H Paris, Paul N Newton

**Affiliations:** a Lao-Oxford-Mahosot Hospital-Wellcome Trust Research Unit (LOMWRU), Microbiology Laboratory, Mahosot Hospital, Vientiane, Lao PDR; b Centre for Tropical Medicine and Global Health, Nuffield Department of Medicine, Churchill Hospital, University of Oxford, Oxford, UK; c Mahidol-Oxford Tropical Medicine Research Unit (MORU), Faculty of Tropical Medicine, Mahidol University, Bangkok, Thailand; d University of the Sunshine Coast, Faculty of Science Health, Education and Engineering, Sippy Downs, Australia; e Queensland Health Forensic and Scientific Service, WHO Collaborating Centre for Reference and Research on Leptospirosis, Brisbane, Australia; f Copenhagen Centre for Disaster Research Department of International Health, Immunology and Microbiology, University of Copenhagen, Copenhagen, Denmark; g UMR_D 190 “Emergence des Pathologies Virales”, Aix-Marseille University, IRD French Institute of Research for Development, EHESP French School of Public Health, Marseille, France

## Abstract

**Background:**

Scrub typhus (caused by *Orientia tsutsugamushi*), murine typhus (caused by *Rickettsia typhi*), and leptospirosis are common causes of febrile illness in Asia; meningitis and meningoencephalitis are severe complications. However, scarce data exist for the burden of these pathogens in patients with CNS disease in endemic countries. Laos is representative of vast economically poor rural areas in Asia with little medical information to guide public health policy. We assessed whether these pathogens are important causes of CNS infections in Laos.

**Methods:**

Between Jan 10, 2003, and Nov 25, 2011, we enrolled 1112 consecutive patients of all ages admitted with CNS symptoms or signs requiring a lumbar puncture at Mahosot Hospital, Vientiane, Laos. Microbiological examinations (culture, PCR, and serology) targeted so-called conventional bacterial infections (*Streptococcus pneumoniae, Neisseria meningitidis, Haemophilus influenzae, S suis*) and *O tsutsugamushi, Rickettsia typhi/Rickettsia* spp, and *Leptospira* spp infections in blood or cerebrospinal fluid (CSF). We analysed and compared causes and clinical and CSF characteristics between patient groups.

**Findings:**

1051 (95%) of 1112 patients who presented had CSF available for analysis, of whom 254 (24%) had a CNS infection attributable to a bacterial or fungal pathogen. 90 (35%) of these 254 infections were caused by *O tsutsugamushi, R typhi/Rickettsia* spp, or *Leptospira* spp. These pathogens were significantly more frequent than conventional bacterial infections (90/1051 [9%] *vs* 42/1051 [4%]; p<0·0001) by use of conservative diagnostic definitions. CNS infections had a high mortality (236/876 [27%]), with 18% (13/71) for *R typhi/Rickettsia* spp, *O tsutsugamushi*, and *Leptospira* spp combined, and 33% (13/39) for conventional bacterial infections (p=0·076).

**Interpretation:**

Our data suggest that *R typhi/Rickettsia* spp, *O tsutsugamushi*, and *Leptospira* spp infections are important causes of CNS infections in Laos. Antibiotics, such as tetracyclines, needed for the treatment of murine typhus and scrub typhus, are not routinely advised for empirical treatment of CNS infections. These severely neglected infections represent a potentially large proportion of treatable CNS disease burden across vast endemic areas and need more attention.

**Funding:**

Wellcome Trust UK.

## Introduction

The most common bacterial pathogens responsible for meningitis in southeast Asia are *Streptococcus pneumoniae, Neisseria meningitidis, Haemophilus influenzae, S suis*, and *Mycobacterium tuberculosis.*^1^ Timely empirical and specific pathogen-directed treatment is essential, usually, except for *M tuberculosis,* including a third-generation cephalosporin. However, many patients with CNS infections do not receive a causal diagnosis despite cerebrospinal fluid (CSF) culture and DNA molecular assays,^2^ partly because of low CSF pathogen density and previous antibiotic use.^2^ Other neglected bacteria probably cause CNS infections in Asia, including pathogens not expected to respond to third-generation cephalosporins. During World War 2, scrub typhus (caused by *Orientia tsutsugamushi*) was a well-recognised cause of lethal meningitis in the Asia-Pacific region, but this clinical experience has largely been forgotten.^3^ Findings of studies in India and Thailand showed that up to 15% of patients with scrub typhus had neurological complications.^4^, ^5^, ^6^ *O tsutsugamushi* DNA was detected in the CSF of Taiwanese patients serologically confirmed to have scrub typhus.^7^ Indeed, altered CNS function is implicit in the name typhus, which means stupor. Similarly, *Rickettsia typhi* (the cause of murine typhus) and other *Rickettsia* species cause meningoencephalitis. Another neglected but common group of pathogens, the *Leptospira* spp have received little attention as causes of CNS infection. In a study in the Philippines, 5% of patients with aseptic meningitis had high serological titres to *Leptospira* spp;^8^ in a Brazilian study, more than 50% of patients with aseptic meningitis were CSF PCR positive for *Leptospira* spp.^9^ However, leptospiral meningitis would be expected to respond to third-generation cephalosporins, if severe leptospirosis does respond to antibiotics.^10^

*Leptospira* and *Rickettsia* species are distributed worldwide^11^, ^12^ and *O tsutsugamushi* is endemic across Asia, the Pacific islands, and northern Australia.^13^ Although a vast human population is potentially exposed to treatable rickettsial and leptospiral diseases, few data exist on the incidence and clinical features of rickettsial and leptospiral CNS infections. Appropriate diagnostic methods or trialled optimum treatments are scarce. Scrub typhus, leptospirosis, and murine typhus are common diseases in Laos, both in the capital, Vientiane, and in rural areas^14^, ^15^ and in adjacent countries, including China and Thailand. Although Asia is geographically, culturally, economically, and ethnically diverse, Laos is an example of the vast areas of rural Asia that are economically poor with little medical information to guide public health policy. Therefore, we assessed whether these pathogens are important causes of CNS infections in Laos.

## Methods

### Study design and participants

In this prospective study, patients were enrolled between Jan 10, 2003, and Nov 25, 2011, at Mahosot Hospital, Vientiane, Laos.^16^ Inpatients of all ages were recruited if a diagnostic lumbar puncture was indicated on the basis of altered consciousness or neurological findings by the attending physicians, and if there were no contraindications. Informed consent (verbal during 2003–06; written during 2006–11) was given by the patient, parents, or guardian (Dubot-Pérès A, et al, unpublished). Ethical approval was granted by OXTREC (University of Oxford, UK) and the Faculty of Medical Sciences Committee (University of Health Sciences, Laos).

### Procedures

Acute encephalitis syndrome and meningitis were defined according to WHO 2003 guidelines.^17^ Acute encephalitis syndrome was defined as the acute onset of fever and either a change in mental status (including symptoms such as confusion, disorientation, coma, or inability to talk) and new onset of seizures (excluding simple febrile seizures) in a person of any age. Meningitis was defined as a sudden onset of fever (>38·5°C rectal or 38·0°C axillary) with one of the following signs: neck stiffness, altered consciousness, or other meningeal signs. If a patient fulfilled criteria for both disorders, we used the term meningoencephalitis. We recorded demographic and clinical data on standardised forms and grouped data according to guidelines.^17^, ^18^ Occupations were classified as farmer, housewife, teacher, government official, driver, building worker, merchant, health worker, police, monk, mechanic, soldier, child (<5 years), schoolboy or girl (5–15 years), student (>15 years), or unemployed.

We measured the CSF opening pressure with manometers. The target CSF volumes were 8 mL for adults (>15 years), 3·5 mL for children (1–15 years), and 2·5 mL for infants (<1 year). We measured CSF lactate and glucose concentrations with Olympus AU400/AU400e Chemistry ImmunoAnalyzers (V-Diagnostic Center, Bangkok, Thailand). Whole blood samples were taken for two blood culture bottles: non-anticoagulated blood for tests on serum and blood clots, and EDTA blood for tests on whole blood, plasma, and buffy coat samples.^14^ CSF and blood cultures were processed as described previously.^14^, ^16^, ^17^, ^19^ The median interval between admission and convalescent serum samples was 10·5 days (range 2–90). We tested for rickettsial antibodies (IgM and IgG) with batched indirect immunofluorescence assays for scrub typhus and murine typhus.^14^ We did leptospiral microscopic agglutination tests in one batch, which were interpreted by the WHO/AO/OIE Collaborating Centre for Reference and Research on Leptospirosis, Brisbane, Australia.^14^ We regarded a four-fold increase between admission and convalescent samples (by immunofluorescence assay or microscopic agglutination test) as evidence of acute infection, and a two-fold increase or decrease (with microscopic agglutination test), a titre of 1:400 or more (with microscopic agglutination test), or a high static titre (≥1:12 800, with immunofluorescence assay) as evidence of probable or recent infection.

*Leptospira* spp (from 2006), *Rickettsia* spp, and *O tsutsugamushi* (from 2008) were cultured as described previously.^14^ In-vitro isolation was attempted from buffy coat for patients with admission-positive murine typhus (ImmunoDot, GenBio, USA) or scrub typhus IgM rapid test (Standard Diagnostics, Korea)^14^ results on serum analysis.

PCR templates were prepared from EDTA buffy coat or CSF samples. We extracted DNA with the QIAGEN DNA Mini kit or QIAGEN EZ-1 extraction-robot.^14^, ^19^ Quantitative PCR assays (qPCR) were done for *O tsutsugamushi, R typhi/Rickettsia* spp, *Leptospira* spp on buffy coat DNA (1 μL) and CSF DNA (5 μL). For the conventional bacterial causes (defined as *Streptococcus pneumoniae, Neisseria meningitidis, Haemophilus influenzae* and *S suis*) qPCRs were done on CSF DNA (3 μL) only. Culture of conventional bacteria was done as described,^19^ *M tuberculosis* by culture on Lowenstein-Jensen media and *Cryptococcal* spp on Sabouraud agar.

*O tsutsugamushi, Rickettsia* spp, *R typhi*, and *Leptospira* spp were retrospectively detected and confirmed by multiple assays in batches (*O tsutsugamushi*: 47 kDa^14^/56 kDa;^20^ *Rickettsia* spp/*R typhi*:^14^, ^21^ 17 kDa/*ompB*^14^; *Leptospira* spp: *LipL32*/*rrs-*conventional^14^, ^22^), or DNA sequencing (Macrogen, Seoul, South Korea) followed by NCBI-BLAST analysis. Established qPCR assays^16^, ^19^, ^23^ for conventional bacterial causes of meningitis were used prospectively on consecutive samples from 2008 and on batched samples from previous years.^16^, ^19^, ^23^

### Statistical analysis

Statistical analysis was done with STATA/IC (version 10). We made comparisons with the χ^2^ (or Fisher's exact test), or Mann-Whitney *U* test, using the conventional bacteria group as the reference group so that factors that might differentiate patients with *R typhi*/*Rickettsia* spp, *O tsutsugamushi*, and *Leptospira* spp infections could be identified. Because several comparisons were made, we report exact p values so that a Bonferroni correction can be applied (α/n, where α=0·05 and n=number of tests), if preferred. We identified independent risk factors for the combined *R typhi/Rickettsia* spp and *O tsutsugamushi* group with logistic regression analysis. Significant variables (p<0·05) from the univariate analysis were included in a multivariate model and we used a stepwise approach to identify predictors. We retained only variables significant at p<0·05 in the final model (appendix p 2). The model was adjusted for presence of eschars. The fit of the models was checked with the Hosmer-Lemeshow goodness-of-fit test and assumptions about linearity with the logit function for continuous variables were confirmed used the link test function in STATA. We defined conservative criteria for diagnosis as pathogen detection by PCR or culture or a four-fold titre rise between admission and convalescent samples.^14^

### Role of the funding source

The funder of the study had no role in study design, data collection, data analysis, data interpretation, or writing of the report. The corresponding author had full access to all the data in the study and had final responsibility for the decision to submit for publication.

## Results

**Figure 1:**
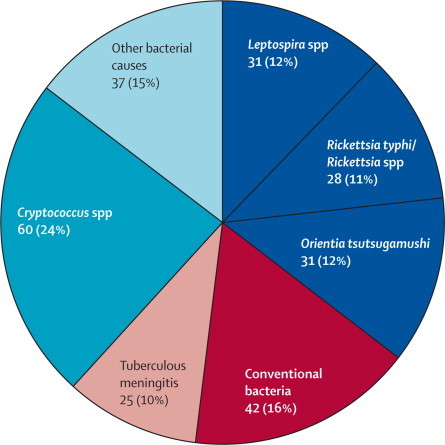
Summary of diagnosed bacterial and fungal infections Conventional bacteria were defined as *Streptococcus pneumoniae, Neisseria meningitidis, Haemophilus influenzae*, or *S suis*.

Between 2003 and 2011, 1112 patients were recruited and physicians collected CSF from 1051 (95%) patients (appendix p 1). Most patients were male and had a history of antibiotic administration (table 1). Of the 1051 patients with CSF samples available, 254 (24%) had evidence of bacterial (194 [76%]) or fungal (60 [24%]) infection, of which 90 (35%) were attributed to *O tsutsugamushi, R typhi*/*Rickettsia* spp, or *Leptospira* spp (figure 1). By PCR, serology, and culture, 45% of inpatients who had a lumbar puncture were assigned a bacterial, fungal, or viral laboratory diagnosis (Dubot-Pérès A, et al, unpublished).

**Table 1:** Clinical and demographic data of patients with suspected CNS infections by pathogen group

	**All patients (n=1112)**	**Conventional bacteria (n=42)**	***Rickettsia typhi* or *Rickettsia* spp (n=28)**		***Orientia tsutsugamushi* (n=31)**		***Leptospira* spp (n=31)**	
				**p value**		**p value**		**p value**
**Demographic data**								
Age (years)	24 (0–85; 1)	14 (0·1–65)	29 (0·3–70)	0·051	16 (0·3–76)	0·412	25 (0·2–72)	0·058
Age <15 years	371/1111 (33%)	21/42 (50%)	7/28 (25%)	0·048	14/31 (45%)	0·813	7/31 (23%)	0·028
Male	696/1111 (63%)	26/ 42 (62%)	18/28 (67%)	1	22/31 (71%)	0·464	22/31 (71%)	0·464
Farmers (if aged >15 years)	110/614 (18%)	6/18 (33%)	2/18 (11%)	0·228	2/13 (15%)	0·412	6/19 (32%)	1
Preadmission antibiotic use	619/992 (62%)	23/36 (64%)	17/25 (68%)	0·790	25/29 (86%)	0·051	17/29 (59%)	0·798
**Symptoms and signs**								
Fever at admission (>38°C)	990/1102 (90%)	41/42 (98%)	27/27 (100%)	1	31/31 (100%)	1	30/31 (97%)	1
Days of fever	4 (0–210; 10)	3 (0-30)	4 (1–30; 1)	0·053	7 (1–15; 1)	0·0001	4 (0–120)	0·144
Headache*	820/932 (88%)	21/25 (84%)	24/25 (96%)	0·349	24/27 (89%)	0·698	28/29 (97%)	0·170
Vomiting	523/1110 (47%)	21/42 (50%)	12/28 (15%)	0·629	18/30 (60%)	0·475	16/31 (52%)	1
Convulsions	334/1108 (30%)	18/41 (44%)	7/28 (25%)	0·132	8/31 (26%)	0·141	6/31 (19%)	0·043
Stiff neck	608/1107 (55%)	29/42 (69%)	14/28 (50%)	0·136	20/30 (67%)	1	15/31 (48%)	0·093
Skin rash	117/1107 (11%)	1/42 (2%)	3/28 (11%)	0·294	6/30 (20%)	0·018	2/31 (7%)	0·571
Hearing loss*	51/931 (6%)	1/25 (4%)	0/25 (0%)	1	3/27 (11%)	0·611	2/29 (7%)	1
Photophobia*	29/982 (3%)	1/41 (2%)	0/28 (0%)	1	4/30 (13%)	0·155	1/31 (3·2)	1
Eschar	21/1105 (2%)	0/42 (0%)	1/28 (4%)	0·400	2/30 (7%)	0·170	0/31 (0%)	1
Tachypnoea†	585/1081 (54%)	25/42 (60%)	20/28 (71%)	0·445	20/31 (65%)	0·808	21/31 (68%)	0·624
Peripheral neurological abnormalities	23/970 (2%)	0/41 (0%)	0/26 (0%)	1	0/22 (0)	1	3/27 (11%)	0·058
GCS	14 (3–15; 61)	13 (3-15; 3)	14 (5-15; 1)	0·442	15 (3–15; 1)	0·013	15 (5–15)	0·274
GCS <15	531/1051 (51%)	26/39 (67%)	14/27 (52%)	0·306	11/30 (37%)	0·016	15/31 (48%)	0·148
Meningitis‡	709/1093 (65%)	33/42 (79%)	19/28 (68%)	0·405	25/31 (81%)	1	21/31 (68%)	0·419
Meningitis (>38·5°C)	403/959 (42%)	25/38 (66%)	7/25 (28%)	0·005	19/28 (68%)	1	14/29 (48%)	0·212
Acute encephalitis syndrome‡	610/1093 (56%)	33/42 (79%)	16/28 (57%)	0·067	15/31 (48%)	0·012	16/31 (52%)	0·023
Meningitis and acute encephalitis syndrome‡	521/1093 (48%)	29/42 (69%)	16/28 (57%)	0·322	14/31 (45%)	0·055	14/31 (45%)	0·055
Died‡	236/876 (27%)	13/39 (33%)	7/26 (27%)	0·784	3/22 (14%)	0·132	3/23 (13%)	0·132
**CSF data**								
Opening pressure (cm H_2_O)§	20 (0–41; 120)	24 (7·8–41; 5)	17 (9-40)	0·162	22 (8–41; 5)	0·610	19 (10–35·5; 2)	0·458
Turbid	145/1000 (15%)	21/40 (53%)	1/25 (4%)	<0·0001	4/25 (16·0)	0·004	3/30 (10%)	<0·0001
Total white cell count (mm^3^)	30 (0–17 200; 104)	410 (0–9600)	10 (0–605; 2)	0·0001	68 (0–653; 5)	0·0001	88 (0–5325; 1)	0·002
Neutrophils (mm^3^)	14·8 (0–17 200; 126)	240 (0–9600)	5·1 (0–545; 2)	0·0001	44·8 (0–535; 6)	0·0004	30 (0–4805; 1)	0·0004
Number of neutrophils ≥1/mm^3^	731/986 (74·1)	39/42 (93%)	17/28 (61%)	0·003	22/25 (88%)	0·664	23/30 (77%)	0·019
Lymphocytes (mm^3^)	10 (0–6976; 134)	25 (0–3515)	5 (0–340; 2)	0·005	21 (0–445; 6)	0·236	21·8 (0–833; 1)	0·352
Number of lymphocytes >5/mm^3^	558/978 (57·1)	32/42 (76%)	10/26 (39%)	0·004	18/25 (72%)	0·775	19/30 (63%)	0·296
Neutrophil to lymphocyte ratio	1 (0–174; 136)	2·7 (0–44; 4)	1 (0–9; 5)	0·0001	1·9 (0·1–19; 9)	0·024	1 (0–9; 4)	0·004
Lactate >4 mmol/L	255/780 (33%)	30/35 (86%)	7/21 (33%)	<0·0001	5/21 (24%)	<0·0001	14/23 (61%)	<0·0001
Glucose <2·5 mmol/L	217/780 (28%)	23/35 (66%)	5/21 (24%)	0·005	3/21 (15%)	<0·0001	7/23 (30%)	0·015
CSF to blood glucose ratio <0·5	380/780 (49%)	32/35 (91%)	8/21 (38%)	<0·0001	9/21 (43%)	<0·0001	8/23 (35%)	<0·0001
Protein >0·4 g/L	600/947 (63%)	32/36 (89%)	10/22 (46%)	0·001	17/22 (77%)	0·278	13/25 (53%)	0·002

Data are median (range; missing) or n/N (%). GCS=Glasgow coma scale. CSF=cerebrospinal fluid. Continuous variables were compared with the Mann-Whitney *U* test and dichotomous variables with the Fisher's exact test. We included only single infections detected either by culture, molecular diagnostic, or four-fold antibody titre rise between admission and convalescent samples. We calculated p values by comparing patients with *Rickettsia typhi* or *Rickettsia* spp, *Orientia tsutsugamushi,* and *Leptospira* spp, with patients with conventional bacteria (p<0·05). For the 19 patients PCR positive for *R typhi* or *Rickettsia* spp group, 16 had *R typhi* but because of insufficient template, we were unable to speciate the other three *Rickettsia* spp.

* Not including children <3 years of age because of difficulty of young children reporting these symptoms.

† >20 breaths per min for adults (>15 years) and variable for children depending on their age.^18^

‡ Including patients discharged moribund, extremely likely to have died at home.

§ The maximum opening pressure that could be measured was 40 cm H_2_O. Pressures >40 cm H_2_O were reported as 41 cm H_2_O.

42 (4%) of 1051 patients had culture or PCR evidence of a monoinfection with a conventional bacterial pathogen: *S pneumoniae* (25 [2·4%]), *N meningitidis* (4 [0·4%]), *H influenzae* (9 [0·9%]), and *S suis* (4 [0·4%]). By comparison, 90 (8·6%) of 1051 patients had culture, PCR, or serological evidence for monoinfections with *O tsutsugamushi, R typhi*/*Rickettsia* spp, or *Leptospira* spp (table 1). The frequency of these combined pathogens (8·6%) was significantly higher than that for conventional bacteria (4·2%; p<0·0001).

Patients with conventional bacterial meningitis were a similar age to those with scrub typhus, but patients with *R typhi* or *Rickettsia* spp and *Leptospira* spp infections tended to be older (table 1). Most (879/1112 [81%]), patients were residents of Vientiane City and Vientiane Province, reflecting the hospital catchment area and was similar for all study groups. No district or occupation was associated with higher patient numbers.

With PCR or culture assays, detection of moninfections with *O tsutsugamushi, R typhi* or *Rickettsia* spp, or *Leptospira* spp infection ranged from 1·9% to 2·9% (table 2). Of 446 patients with data available for both direct and serological results, we detected evidence of scrub typhus, *Rickettsia* spp or murine typhus, or leptospirosis in 59 (13%) of 446 patients. *Leptospira interrogans* was the most commonly identified *Leptospira* species by microscopic agglutination test or sequencing (GenBank KJ150298-KJ150302; appendix p 3). We identified *Rickettsia* spp as *R typhi* for 16 (84%) of 19 patients who were qPCR positive, but for three patients the *Rickettsia* species could not be established because no template remained. In addition to the 90 patients with *O tsutsugamushi, R typhi/Rickettsia* spp and *Leptospira* spp monoinfections, we recorded grade 1 and grade 2 multiple infections in an additional 14 patients (appendix p 4).^24^

**Table 2:** Overview of diagnostic findings by disease groups and detection method, excluding patients with evidence of grade 1 or grade 2 co-infections

	**Conventional bacteria**	***Rickettsia* spp or *R typhi***	***Orientia tsutsugamushi***	***Leptospira* spp**
**Direct detection**				
PCR (CSF)	42/1051 (4·0%)	15/983 (1·5%)	20/1011 (2·0%)	6/1014 (0·6%)
PCR (blood)	NA	4/509 (0·8%)	16/515 (3·1%)	6/509 (1·2%)
Culture (CSF)	12/1051 (1·0%)	NA	NA	NA
Culture (blood)*	8/1051 (0·8%)	1/62 (1·6%)	3/62 (4·8%)	2/646 (0·3%)
Total PCR/culture	42/1051 (4·0%)	19/1051 (1·9%)	30/1,051 (2·9%)	13/1051 (1·2%)
**Serology (IFA/MAT; plasma)**				
Evidence of acute infection	NA	9/795 (1·1%)	8/795 (1·0%)	20/541 (3·7%)
Probable or recent infection	NA	0/795 (0·0%)	11/795 (1·4%)	24/541 (4·4%)
Four-fold rise/PCR/culture	NA	28/1051 (2·7%)	31/1051 (2·9%)	31/1051 (2·9%)

Data are the number of positive samples by laboratory investigation (percentages of positives) for the different pathogens. Patients who were positive by more than one method are included for the individual methods but the total is given for infected patients rather than samples. Seroconversion was defined as a four-fold antibody titre rise between admission and convalescent sample, while a high static titre (≥1:12 800) was deemed evidence for infection. CSF=cerebrospinal fluid. IFA=immunofluorescence assay. MAT=microscopic agglutination test.

* Mahosot Hospital Microbiology Laboratory participates in the UK NEQAS General Bacteriology and Antimicrobial Susceptibility Testing scheme.

**Figure 2:**
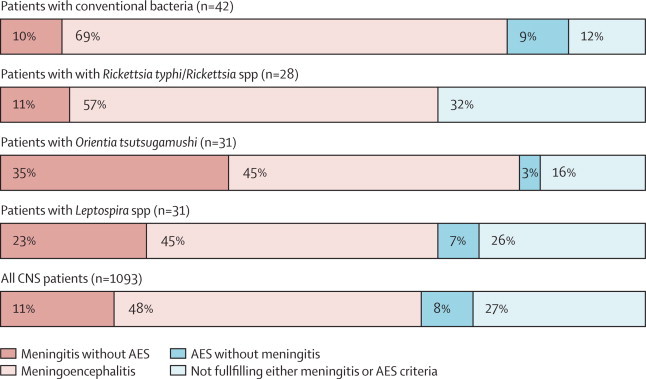
Patients who fulfilled WHO criteria^17^ for meningitis, meningoencephalitis, AES, or none of these criteria Meningoencephalitis is defined as fulfilling criteria for both meningitis and AES. AES=acute encephalitis syndrome.

More than half of all patients met the WHO criteria for meningitis (709/1093 [65%]) or acute encephalitis syndrome (610 [56%]), 84 (74%) met either criteria, and 521 (48%) fulfilled both criteria (table 1 and figure 2). Patients with a conventional bacterial CNS infection presented with the shortest median duration of fever (p=0·005), the highest frequencies of convulsions (p=0·023) and neck stiffness (p=0·182), the lowest median Glasgow coma scale (GCS) scores on admission (p=0·062), and the highest mortality (p=0·076), compared with patients with *R typhi* or *Rickettsia* spp and *Leptospira* spp infections (table 1).

Patients with a CNS infection caused by *R typhi*/*Rickettsia* spp tended to present later in their illness than patients with conventional bacteria (table 1). They had the lowest frequency of vomiting and none reported photophobia. The mortality of patients with *R typhi* or *Rickettsia* spp (27%) was nearly double that of patients with scrub typhus (14%, p=0·307) and leptospirosis (13%, p=0·299); however, these differences were not significant. Notably, the mortality of patients with *R typhi*/*Rickettsia* spp was similar to those infected with conventional bacteria (about 33%). Furthermore, compared with patients with conventional bacteria, a similar proportion of patients presented with a reduced GCS score (table 1).

Patients with *O tsutsugamushi* also presented late in their illness and had a high frequency of rash (20%; table 1). 81% fulfilled the WHO meningitis criteria (figure 2) with a significantly higher median GCS score and the highest reported preadmission antibiotic use (86%) of investigated groups.

Fewer patients in the *Leptospira* group had convulsions (19%) than those in the conventional bacterial group (44%) and all other investigated groups, but presented with the highest frequency of peripheral neurological abnormalities (11%). One patient had GCS 15/15 and bilateral limb weakness (Medical Research Council [MRC] power 2/5) without knee or ankle reflexes and unrecorded sensation; one had GCS 15/15 and bilateral limb weakness (MRC power 4/5) with unrecorded reflexes and sensation; and a third had GCS 9/15, convulsions, and right leg weakness (MRC power 4/5) with normal reflexes and unknown sensation.

Patients with *O tsutsugamushi* or *R typhi* or *Rickettsia* spp infections presented with significantly longer fever duration (median 6 days, range 1–30 *vs* 4 days, 0–120; p=0·004), and significantly more commonly had a rash (9/58 with *O tsutsugamushi* or Rickettsia spp and 3/73 with Leptospira spp or conventional bacteria infection; p=0·033) compared with patients with conventional bacteria and leptospira. In multivariate analysis, we identified no independent clinical risk factors for *O tsutsugamushi* or *R typhi*/*Rickettsia* spp infections. The mortality in this combined group (10/48 [21%]) was not significantly different from patients with conventional bacterial infection (p=0·189).

Patients with conventional bacterial infections had high opening pressures, frequent CSF turbidity, and high cellularity. Cells were mainly neutrophils, with high CSF lactate, low glucose, and high protein concentrations (table 1).

Despite similar clinical severity and mortality, the CSF characteristics of patients infected with *R typhi* or *Rickettsia* spp differed from the conventional bacteria group. The opening pressure tended to be lower and only one patient had CSF turbidity. CSF cellularity, neutrophil to lymphocyte ratio, and lactate and protein concentrations were also significantly different from conventional bacterial infections (table 1).

For patients infected with *O tsutsugamushi***,** opening pressures were similar to those with conventional bacterial illness, but CSF turbidity and cellularity were significantly less common and CSF lactate concentrations were the lowest of all groups (table 1). Compared with those with *R typhi* or *Rickettsia* spp infections, patients with scrub typhus had significantly higher CSF white cell counts (p=0·018) and a two-fold higher neutrophil to lymphocyte ratio (p=0·045).

For patients infected with *Leptospira* spp, the CSF opening pressure was similar to the other groups, but CSF turbidity and cellularity were rare, with lower protein and higher glucose concentrations than patients in the conventional bacteria group (table 1).

Visual turbidity (5/50; p=0·002) and cellularity (median 38·5 cells per mm^3^, range 0–653; p=0·0001) were significantly less frequent for patients with *O tsutsugamushi* or *R typhi*/*Rickettsia* spp infections, than for patients with *Leptospira* spp or conventional bacterial infections (turbidity 24/70; median cellularity 235 cells per mm^3^, range 0–9, 600). Compared with *O tsutsugamushi* or *R typhi*/*Rickettsia* spp infections (median neutrophil to lymphocyte ratio 1·0, range 0–19), patients with conventional bacteria and *Leptospira* spp infections had two times higher neutrophil to lymphocyte ratios (median ratio 2, range 0–174; p=0·011). Eight (19%) of 42 patients with *O tsutsugamushi* or *R typhi*/*Rickettsia* spp infections presented with CSF glucose lower than 2·5 mmol/L compared with 30 of 51 patients in the combined *Leptospira* spp and conventional bacterial infection group. 12 (29%) of 42 patients with *O tsutsugamushi* or *R typhi*/*Rickettsia* spp infections had lactate concentrations higher than 4 mmol/L compared with 39 (67%) of 58 patients in the combined *Leptospira* spp and conventional bacterial infection group (p<0·0001). A lower white cell count was associated with an increased odds of *O tsutsugamushi* or *R typhi*/*Rickettsia* spp infection; the odds decreased 1% with every unit increase in white cell count (OR 0·997, 95% CI 0·995–0·999).

**Figure 3:**
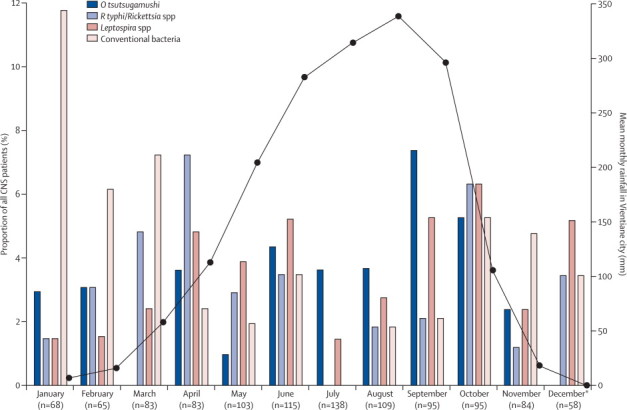
Monthly proportions of CNS patients diagnosed with *Orientia tsutsugamushi, Rickettsia typhi/Rickettsia* spp, *Leptospira* spp, or so-called conventional bacterial infections among all patients recruited with suspected CNS disease, 2003–11 Total number of patients recruited per month during this study, 2003–11, is shown by bars corresponding to the indicated pathogens. The line represents the mean rainfall (mm) per month in Vientiane during the investigated years. Data from Department of Meteorology and Hydrology, Ministry of Natural Resources and Environment, Lao PDR. *Patient recruitment to this study stopped in November, 2011.

Infections with conventional bacteria showed a distinct seasonal pattern, peaking in the dry (November–April) season (dry: 26/441 [6%], wet: 15/655 [2%], p=0·002; figure 3). By contrast, *O tsutsugamushi* was diagnosed with greater frequency in the wet season (27/655 [4%]) than the dry season (9/441 [2%], p=0·055). The frequency of *R typhi*/*Rickettsia* spp and *Leptospira* spp did not significantly differ between seasons (*R typhi*/*Rickettsia* spp; dry: 17/655 [3%]; wet: 16/441 [4%], p=0·369; *Leptospira* spp; dry: 26/655 [2%]; wet: 13/441 [3%], p=0·371).

17 (55%) of 31 patients with *O tsutsugamushi* were treated with appropriate antibiotics compared with 11 (39%) of 28 with *R typhi*/*Rickettsia* spp infections (table 3). Ten (17%) of 59 patients with *O tsutsugamushi* or *R typhi*/*Rickettsia* spp infection died, of whom three received appropriate treatment. In this small sample, we noted no significant association between patients receiving appropriate treatment and outcome (p=0·179). Nearly all (30/31) patients with a *Leptospira* spp infection received appropriate treatment with doxycycline, chloramphenicol, a cephalosporin, or penicillin alone (14/30) or in combination (16/30). All three patients of the 24 patients with fatal leptospirosis received appropriate treatment.

**Table 3:** Treatment received by patients with *Orientia tsutsugamushi* or *Rickettsia typhi* or *Rickettsia* spp monoinfections

		***Orientia tsutsugamushi* (n=31)**	***Rickettsia typhi* or *Rickettsia* spp (n=28)**
Appropriate treatment*		17	11
	Doxycycline	14	9
	Chloramphenicol	2	2
	Rifampicin†‡	1	..
Inappropriate treatment		14	17
	Ceftriaxone	6	7
	Ceftriaxone or combination§	5	3
	Other‡¶	1	3
Unknown or not treated		2	4

* Intravenous azithromycin, doxycycline, and tetracycline are not available in Laos.

† Received as part of four fixed-dose combination antituberculosis treatment (4FDC).

‡ Whether rifampicin is effective for murine typhus is unknown.

§ Combination drug: penicillin (n=2), gentamicin (n=2), gentamicin and ciprofloxacin (n=1), ceftazidime plus ofloxacin plus levofloxacin (n=1).

¶ Penicillin alone (n=2), sulfonamides or antifungal treatment (n=1), amphotericin B/4FDC (n=1).

## Discussion

These data suggest that *O tsutsugamushi, R typhi* or *Rickettsia* spp, and *Leptospira* spp infections are the leading causes of bacterial CNS infections in Laos. With increasing evidence that *O tsutsugamushi, R typhi* or *Rickettsia* spp, and *Leptospira* spp are important causes of fevers, these findings raise concerns that these infections are responsible for a large proportion of neglected but treatable CNS disease burden in the many endemic countries and in travellers (panel).^12^, ^14^, ^25^, ^26^

Panel **Research in context**

**Systematic review**

We searched PubMed for relevant articles published in English up to April, 2014, using the search terms “central nervous system infections” or “meningitis” or “encephalitis” together with “scrub typhus” or “*O tsutsugamushi”*, “murine typhus” or ”Rick*”, or ”leptospir*”. 195 relevant publications were identified, describing case series and case reports of CNS disease caused by these pathogens with some including clinical and cerebrospinal fluid characteristics or both. However, none of the published scientific literature described the prospective investigation of consecutive hospital patients to assess the contribution of scrub typhus, murine typhus, and leptospirosis to the CNS disease burden in endemic areas. Articles published in PubMed before 1966 alluded to the potential of *Orientia tsutsugamushi, Rickettsia* spp, and *Leptospira* spp to cause CNS disease and underlined the need for further investigation.

**Interpretation**

Our findings show that rickettsial and leptospiral pathogens are important causes of meningitis, encephalitis, and meningoencephalitis in Laos. Our findings show the importance of these neglected but important diseases and suggest that clinicians and microbiologists need to be aware of these treatable causes of severe CNS infection. In view of the ineffectiveness of the standard empirical cephalosporin and penicillin treatments against rickettsial pathogens, these data emphasise the need to rethink treatment guidelines in endemic regions to consider including an antirickettsial antibiotic.

8% of all CNS infections and 46% of identified bacterial causes were attributable to *O tsutsugamushi, R typhi*/*Rickettsia* spp, or *Leptospira* spp, with conservative and robust diagnostic definitions; more than double the 22% attributed to four conventional bacteria species. The overall mortality of CNS infections in Laos was 27%, with group-specific mortality of 18% for *Orientia, Rickettsia*, and *Leptospira* spp, and 33% for conventional bacteria, which emphasises the importance of improving diagnostic and treatment strategies. Comparisons of clinical findings highlight differences that might serve as diagnostic clues. For example, patients with *R typhi*/*Rickettsia* spp and *Leptospira* spp infections were older and patients with *O tsutsugamushi* presented later in their illness, consistent with reports from India,^6^ and commonly had skin rashes. Elsewhere, hearing loss has often been noted in patients with *O tsutsugamushi*,^27^ but was not significantly more common in patients with *O tsutsugamushi* and CNS disease in Laos than other patient groups. Consistent with previous findings,^6^ patients with *O tsutsugamushi* and *R typhi/Rickettsia* spp infections presented with low, but abnormal CSF white cell counts; turbid CSF and raised CSF lactate concentrations were infrequent.

Data from recent reports suggest that admission interstitial pneumonitis is associated with meningitis in patients with *O tsutsugamushi* infection.^6^, ^28^ Although most Lao patients with *O tsutsugamushi* presented with tachypnoea, this was not significantly more frequent than in other groups (p=0·587). Furthermore, *O tsutsugamushi* infection commonly presents with tachypnoea without CNS involvement.^15^ *R typhi/Rickettsia* spp infections were more severe than *O tsutsugamushi* and leptospirosis in terms of GCS and mortality. However, *R typhi* infection is generally regarded as benign^29^ and although it is distributed worldwide, is rarely included in the differential diagnosis of CNS disease.

Reduced consciousness and seizures were the most common neurological symptoms in Indian patients with neuroleptospirosis.^30^ We did not record this finding in Laos, which raises questions about variation in strain virulence and host susceptibility. Three patients with leptospirosis had some neurological evidence of intracerebral lesions, consistent with the putative association between leptospirosis and Moyamoya disease, but cerebral angiography, which is not available in Laos, is needed to confirm the diagnosis.^31^ Notably, only patients with leptospirosis had abnormal peripheral neurological symptoms and signs.

Conventional bacterial infections were significantly associated with turbid, cellular CSF containing high neutrophil counts. The high frequency of CSF abnormalities (including increased white cell counts, turbidity, and lactate concentrations) in all patients who had a lumbar puncture, but without a causal diagnosis, suggests that many of these patients had undetected infections and that patients with important CNS pathology are not receiving lumbar punctures.

During this 9 year investigation, we noted a distinct seasonal pattern, similar to findings for non-malarial fevers in Laos.^14^ Conventional bacterial infections (eg, *N meningitidis* and *S pneumoniae),* were most frequent in the dry season, peaking in January, consistent with data from India,^32^ whereas *Orientia, Rickettsia*, and *Leptospira* species were detected in nearly 20% of febrile patients diagnosed with CNS disease at the end of the rainy season. This seasonality and CSF characteristics could help to guide clinicians' differential diagnosis.

Our study has important limitations, including the use of suboptimum samples for leptospiral culture,^33^ the known limitations of rickettsial diagnostics,^34^, ^35^ and the use of stored samples. However, these limitations probably led to the underestimation of the incidence of *Leptospira* spp, *O tsutsugamushi,* and *R typhi*/*Rickettsia* spp infections. The widespread use of over-the-counter antibiotics^2^ before admittance to hospital probably reduced culture rates for conventional bacteria, and we did not do serology assays for these pathogens. The lack of clustered results and stringent sample handling protocols, physical separation of processes, and the use of uracil-DNA glycosylase in the PCR mix, makes specimen contamination very unlikely. The three *Rickettsia* spp infections that could not be speciated were probably *R typhi* because this is the main species in Laos.^14^, ^36^

Our data suggest that empirical treatment practice for CNS infections in Laos, where third-generation cephalosporin monotherapy is generally used, should be reconsidered. Although current guidelines are probably effective for leptospiral CNS disease, there is no evidence for their efficacy against *O tsutsugamushi* and *R typhi*/*Rickettsia* spp, which should be treated with doxycycline or chloramphenicol. Azithromycin or rifampicin might be active against *O tsutsugamushi* CNS disease.^37^ More data are needed for CSF drug levels in patients with typhus and variability in minimum inhibitory concentrations against *R typhi*/*Rickettsia* spp and *O tsutsugamushi,* with clinical trials to inform optimum treatment.^28^ Because of the paucity of accessible and accurate admission laboratory typhus diagnostics,^34^, ^38^ empirical treatment with doxycycline plus a third-generation cephalosporin might be appropriate in areas endemic for scrub typhus and murine typhus. However, combination of bacteriostatic tetracyclines with bactericidal cephalosporins might reduce the efficacy in typhus-endemic areas for treatment of conventional bacterial pathogens, such as *S pneumoniae.*^39^ The optimum management of patients with either confirmed *O tsutsugamushi, R typhi*/*Rickettsia* spp, or *Leptospira* spp CNS infection or of those in which these or conventional bacteria are suspected remains unclear, with little evidence to guide policy. Indeed, there is very little evidence on the pharmacokinetics of the tetracyclines in CSF, or optimum dose for CNS disease.^40^ These findings suggest that greater appreciation and further investigation of *Orientia, Rickettsia,* and *Leptospira* spp as neglected but treatable causes of CNS disease in other endemic areas globally is urgently needed.^3^, ^41^ Although pathogen discovery has an important role, we suggest that optimising diagnosis, treatment, and prevention of these neglected but common bacteria might have a more rapid beneficial public health outcome.
