# Supplementary material for: Orientia, rickettsia, and leptospira pathogens as causes of CNS infections in Laos: a prospective study
Source: Lancet Glob Health. 2015 Feb;3(2):104–12. doi: 10.1016/S2214-109X(14)70289-X (PMC4547322; doi:10.1016/S2214-109X(14)70289-X)
Supplement: Supplementary appendix [file mmc1.pdf]

THE LANCET Global Health

Supplementary appendix

This appendix formed part of the original submission and has been peer reviewed. We post it as supplied by the authors.

Supplement to: Dittrich S, Rattanavong S, Lee SJ, et al. Orientia, rickettsia, and leptospira pathogens as causes of CNS infections in Laos: a prospective study. *Lancet Glob Health* 2015; **3:** e104–12.

Appendix Figure 1. Flow chart of patient recruitment and samples available for laboratory tests.

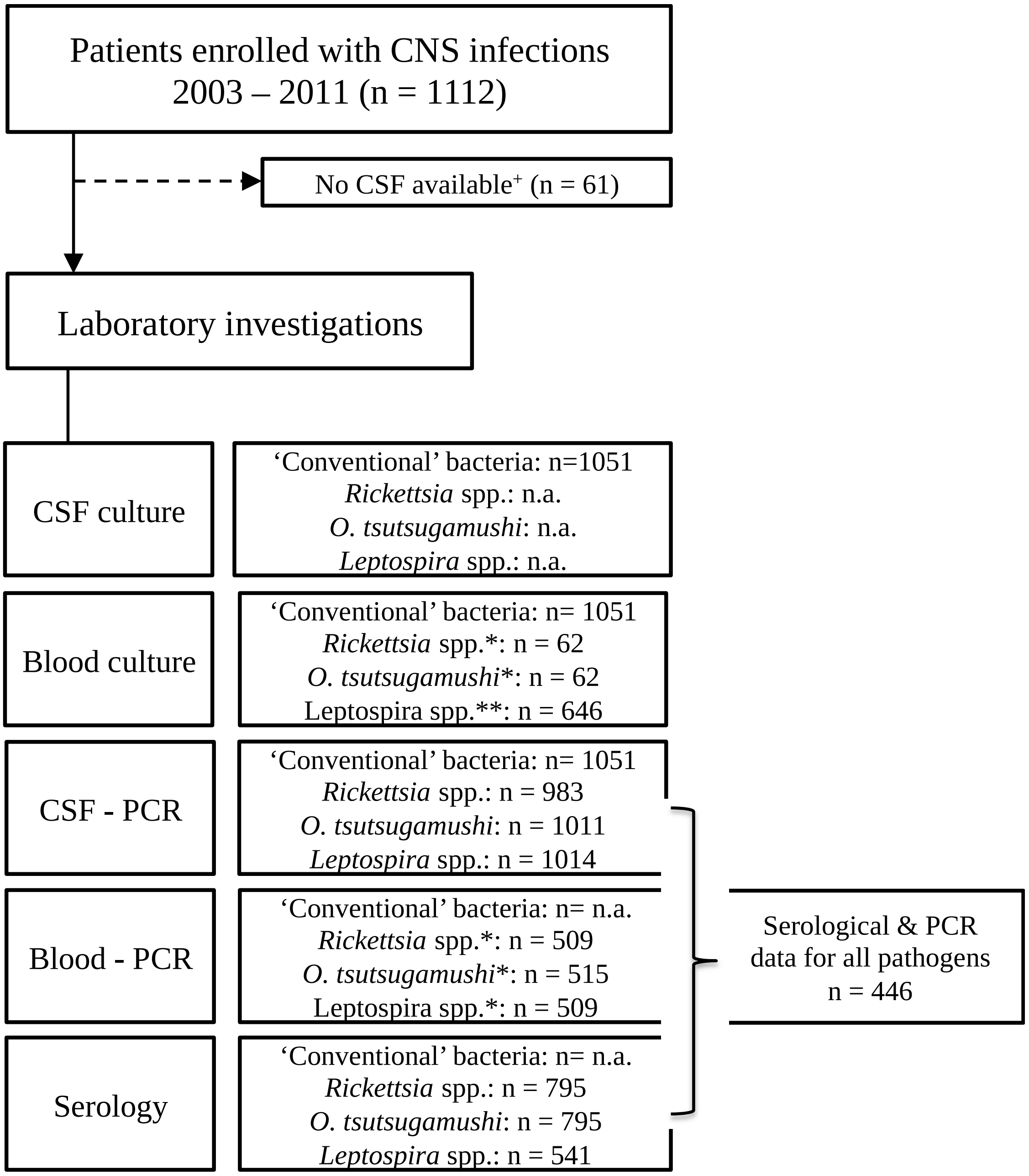

n.a. = not available
+ LP attempted, CSF collection not possible.
* Buffy coat was used for seeding culture and/or as PCR template.
** Blood clot was used for seeding culture and/or as PCR template

Appendix Table 1: Overview of variables included in the univariant analysis.
Continuous variables are expressed as median (range; missing) and compared using the Mann-Whitney U test, while dichotomous variables are expressed as number/total (%) and were compared using Fisher's exact test. Leptospira and 'conventional' bacteria have been combined as they are expected to respond to cephalosporins whereas *R. typhi/Rickettsia* spp. and *O. tsutsugamushi* are not

	R. typhi/*Rickettsia* spp./ *O. tsutsugamushi*	*Leptospira* spp./ 'Conventional' bacteria	p-value
	n=59	n=73	
Age < 15 years	21/59 (39·6)	28/73 (38·4)	0·856
Pre-admission antibiotic use	42/54 (77·8)	40/165 (24·2)	0·074
**Symptoms & signs**			
Days of fever	6 (1-30; 2)	4 (0-120)	**0·004**
Headache**	48/52 (92·3)	49/54 (90·7)	1
Vomiting	30/58 (51·7)	37/73 (50·7)	1
Convulsions	15/59 (25·4)	24/72 (32·9)	0·344
Stiff neck	34/58 (58·6)	44/73 (60·3)	0·860
Skin rash	9/58 (15·5)	3/73 (4·1)	**0·033**
Hearing loss**	3/52 (5·8)	3/54 (5·6)	1
Photophobia**	4/58 (6·9)	2/72 (2·8)	0·406
Eschar	3/58 (5·2)	0/73 (0)	0·084
Tachypnea[#]	40/59 (67·8)	46/73 (63·0)	0·587
Peripheral neurological abnormalities[+]	0/48 (0)	3/68 (4·4)	0·266
GCS	15 (3-15; 2)	14 (3-15; 3)	0·117
Meningitis[§]	44/59 (74·6)	54/73 (74·0)	1
Meningitis (>38·5˚C)	26/53 (49·1)	39/67 (58·2)	0·359
AES[§]	31/59 (52·5)	49/73 (67·1)	0·108
**Cerebrospinal fluid data**			
Opening pressure (cmH_2O)[++]	20 (8-41; 5)	21 (7·8-41; 7)	0·847
Turbid	5/50 (10)	24/70 (34·3)	**0·002**
Total white cell count/mm^3	38·5 (0-653; 7)	235 (0-9,600; 1)	**0·0001**
Neutrophil:Lymphocyte ratio	1 (0-19; 14)	2 (0-174·4; 8)	**0·011**
Lactate >4 mmol/L	12/42 (28·6)	39/58 (67·2)	**<0·0001**
Glucose <2·5 mmol/L	8/42 (19·0)	30/58 (51·7)	**0·001**
CSF:blood glucose ratio <0·5	17/42 (40·5)	40/58 (69·0)	**0·007**
Protein >0·4 g/L	27/44 (61·4)	45/61 (73·8)	0·205

**Children <3 years of age, were excluded from the analysis of clinical symptoms as they are difficult to assess by a third person and cannot be reliably described by the child themselves.
[#]Respiratory rates >20 breath/minutes was considered abnormal for adults (>15years) and for children depending on their age as defined[19].
[+] Peripheral neurological abnormalities: Patient 1/ GC3 15/15, bilateral limb weakness (Medical Research Council (MRC) power 2/5) without knee or ankle reflexes and unrecorded sensation; 2/ GCS 15/15, bilateral limb weakness (MRC power 4/5) with unrecorded reflexes and sensation; 3/ GCS 9/15, convulsions, right leg weakness (MRC power 4/5) with normal reflexes and unknown sensation
'Acute Encephalitis Syndrome' (AES) was defined according to WHO guidelines 'as a person of any age, at any time of year with the acute onset of fever and either a change in mental status (including symptoms such as confusion, disorientation, coma, or inability to talk) and/or new onset of seizures (excluding simple febrile seizures)'. 'Meningitis' was defined according to WHO guidelines as 'a sudden onset of fever (>38·5°C rectal or 38·0°C axillary) with one of the following signs: neck stiffness, altered consciousness or other meningeal sign(s)'.
[++]The maximum opening pressure that could be measured was 40 cm H_2O. Pressures higher than 40 cm H_2O were reported as 41 cm H_2O.

Appendix Table 2: Overview of *Leptospira* spp. and serovar determined by MAT.
In nine patients serological evidence for two serotypes was found and in one patient, evidence for three serotypes was found by MAT. For two samples, sequencing and MAT data were available, one being identified by both methods as *Leptospira interrogans (MAT:* serovar Copenhageni) while the other sample was typed as *Leptospira kirschneri* serovar Canicola by MAT and *Leptospira interrogans* by sequencing.

Serotyping (MAT)	n
Leptospira interrogans serovar Copenhageni	11
Leptospira interrogans serovar Hebdomadis	10
Leptospira interrogans serovar Mwalok	8
Leptospira kirschneri serovar Canicola	6
Leptospira interrogans serovar Hardjo	5
Leptospira borgpetersenii serovar Javanica	3
Leptospira interrogans Autumnalis	4
Leptospira borgpetersenii serovar Ballum	2
Leptospira kirschneri serovar Grippotyphosa	2
Leptospira interrogans serovar Australis	1
Leptospira weilii serovar Celledoni	1
Leptospira kirschneri serovar Cynopteri	1
Leptospira noguchii serovar Djasiman	1
Leptospira noguchii serovar Panama	1

Appendix Table 3. Number of patients with Grade I and Grade 2 co-infections[24]; 14 patients had more than one infection detected.

Pathogen	*O. tsutsugamushi*	*R. typhi* or *Rickettsia* spp.	*Leptospira* spp.
O. tsutsugamushi	31*	-	-
R. typhi/Rickettsia spp.	0	28*	-
Leptospira spp.	4	0	31*
S. suis	0	1	0
*S. viridans***	0	1	0
S. aureus	0	0	1
B. pseudomallei	0	0	1
Cryptococcus spp.	0	1[++]	1
Triple infections[+]	1	2	1

* Single infections (diagnosis based on PCR, culture and 4 fold rise in titre for IFA and MAT)
** Probable culture contamination
[+] Triple infections: patient 1- *R. typhi* (IFA) & *Leptospira* spp. (MAT) & *B. pseudomallei* (culture); patient 2 - *R. typhi* (PCR) & *O. tsutsugamushi* (IFA) *& Mycobacterium tuberculosis* (culture); patient 3- *O. tsutsugamushi* (PCR) & *R. typhi* (PCR) & *Salmonella enterica* group D (culture); patient 4: *Leptospira* spp. (PCR) & *E. coli* (culture) & *Edwardsiella tarda (*culture)
++ this patient was reported in Dittrich *et al.* (2014)[36]
